# Cecal Microbial Hydrogen Cycling Potential Is Linked to Feed Efficiency Phenotypes in Chickens

**DOI:** 10.3389/fvets.2022.904698

**Published:** 2022-06-21

**Authors:** Gustavo Antonio Ramírez, Jitendra Keshri, Isabella Vahrson, Arkadiy I. Garber, Mark E. Berrang, Nelson A. Cox, Fernando González-Cerón, Samuel E. Aggrey, Brian B. Oakley

**Affiliations:** ^1^College of Veterinary Medicine, Western University of Health Sciences, Pomona, CA, United States; ^2^Leon H. Charney School of Marine Sciences, Haifa University, Haifa, Israel; ^3^School of Life Sciences, Arizona State University, Tempe, AZ, United States; ^4^Poultry Microbiological Safety and Processing Research Unit, USDA Agricultural Research Service, Athens, GA, United States; ^5^Departamento de Zootecnia, Chapingo Autonomous University, Estado de Mexico, Mexico; ^6^NutriGenomics Laboratory, Department of Poultry Science, University of Georgia, Athens, GA, United States

**Keywords:** microbiome, poultry, microbiome transplant, hydrogen, archaea, methanogens, agriculture, metagenome

## Abstract

In chickens, early life exposure to environmental microbes has long-lasting impacts on gastrointestinal (GI) microbiome development and host health and growth, via mechanisms that remain uncharacterized. In this study, we demonstrated that administrating a fecal microbiome transplant (FMT) from adults to day-of-hatch chicks results in significantly higher body mass of birds and decreased residual feed intake (RFI), implying enhanced feed efficiency, at 6 weeks of age. To assess the potential mechanisms through which FMT affects adult bird phenotype, we combined 16 S rRNA gene amplification, metagenomic, and comparative genomic approaches to survey the composition and predicted activities of the resident microbiome of various GI tract segments. Early life FMT exposure had a long-lasting significant effect on the microbial community composition and function of the ceca but not on other GI segments. Within the ceca of 6-week-old FMT birds, hydrogenotrophic microbial lineages and genes were most differentially enriched. The results suggest that thermodynamic regulation in the cecum, in this case via hydrogenotrophic methanogenic and sulfur-cycling lineages, potentially serving as hydrogen sinks, may enhance fermentative efficiency and dietary energy harvest capacity. Our study provides a specific mechanism of action through which early-life microbiome transplants modulate market-relevant phenotypes in poultry and, thereby, may represent a significant advance toward microbiome-focused sustainable agriculture.

## Introduction

The poultry gastrointestinal (GI) microbiome metabolizes polysaccharides (1, 2), regulates immunity (3), and provides energy as amino acids and short-chain fatty acids (SCFAs) (4, 5). It is therefore a key determinant of developmental outcomes for all animals, including those of agricultural importance (6). Over the last decade, the poultry GI microbiome has increasingly become recognized as a functional system of the animal itself and a focal component of poultry husbandry influencing bird health, productivity, and food safety (7–9). However, despite recent progress, a mechanistic understanding of how the composition and activities of complex GI microbial communities affect agriculturally desirable phenotypes remains incomplete for poultry.

The primary energy sources for commercial chickens are plant-derived carbohydrates. Unlike readily digestible starches, non-starch polysaccharides (NSPs) may pass undigested to the caecum because of their high structural variability as an enzymatic substrate (10). The ceca, paired blind pouches emerging at the junction of the ileum and colon, are anoxic microbial habitats that host the highest microbial loads and species diversity in the chicken GI tract and are the primary site for carbohydrate fermentation into short-chain fatty acids (SCFAs) and gases (11, 12). Other functions performed by this understudied organ include water and electrolyte exchange (13) and, based on the presence of cecal tonsils containing avian immunoglobins as well as B and T cells, non-digestion related immunological roles (14, 15). The plant-based diets of fowl have led to evolutionarily selection for large ceca (16). Accordingly, the ceca may represent an important symbiotic organ for the targeted modulation of microbial energy harvest from the diet and certain host phenotypes.

Recently, we reported that administering a fecal microbiome transplant (FMT) to newly hatched chickens elicits long-lasting microbial community changes in the ceca that are associated with bird phenotypic changes later in life (17). These results suggest that, through modulation of specific cecal taxa, key metabolic pathways can be optimized, potentially leading to enhanced community fermentative performance that physiologically manifests as improved host phenotypes. Herein, we take a ‘top-down' approach, starting with significant differences in market age (6-week-old) bird performance induced by a day-of-hatch FMT and then use metagenomic and gene targeted surveys to identify specific lineages and metabolic pathways associated with these phenotypes. Noting that fermentations i) invariably produce hydrogen gas while replenishing electron carriers (18) and ii) are less thermodynamically favorable as the partial pressure hydrogen gas increases (19), we explicitly consider the role of hydrogen cycling as an overarching modulatory mechanism for cecal fermentation efficiency.

## Results and Discussion

### FMT Induced Significant Differences in Bird Phenotype

**Figure 1:**
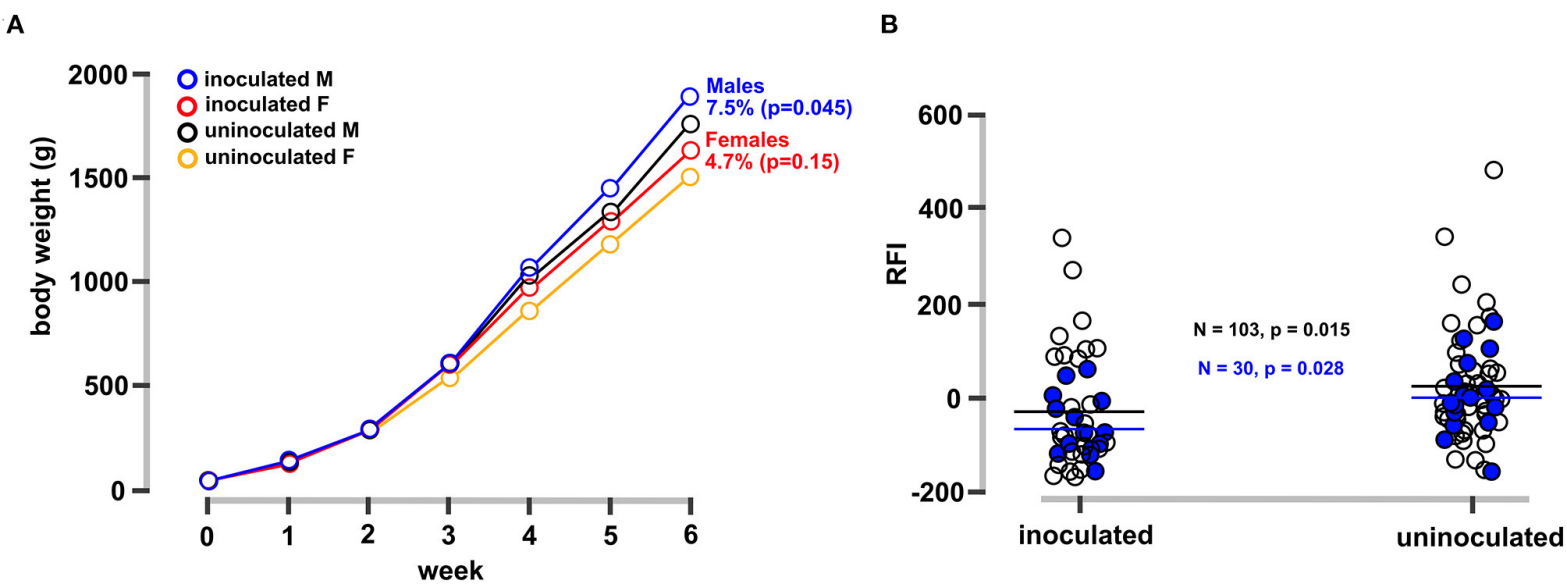
Phenotypes of FMT-inoculated vs. uninoculated control chickens. **(A)** Average body weight for inoculated and uninoculated male and female bird groups over a 6-week period. Chicks received a single inoculum at day of hatch and were reared in identical conditions as described in the Methods section. Groups with letter M and F refer to male and female chicks. **(B)** Residual feed index (RFI, actual feed intake relative to expected; lower values represent increased efficiency) for each experimental group. Black horizontal bars depict the mean of all observations and blue horizontal bars depict the mean for birds sampled for metagenomic sequencing. Differences of means were significant by Kruskal-Wallis or student's *t*-test as indicated on the figure.

FMT-inoculated chicks showed significant improvements in body weight gain (Figure 1A) and feed efficiency (Figure 1B) 6 weeks after inoculation. Inoculated male and female groups gained an average of 7.5 and 4.5%, respectively, more weight at 6 weeks of age relative to controls (Figure 1A). In addition to weight gain, residual feed intake (RFI) is another important metric for assessing feed costs and optimizing poultry production (20). There are several quantitative trait loci (QTL) associated with RFI (21) and duodenal transcriptomics has identified genes related to digestibility, metabolism, and biosynthesis by differential expression analysis between low and high RFI groups (22, 23). Here, in addition to increased weight gain (Figure 1A), mean RFI values were significantly lower (more efficient) in the FMT-inoculated compared to the non-inoculated chickens (Figure 1B).

### Overview of Ileal, Jejunal, and Cecal Microbial Community Profiles

A 16S rRNA gene ordination survey of microbial communities across ileum, jejunum, and cecal GI segments showed that ileum and jejunum communities from both experimental groups were highly similar to each other, in contrast to cecal communities that differed significantly according to FMT administration (Supplementary Figure S1), corroborating the separation of hindgut and foregut microbiota previously reported for chickens (24). Significant differences between experimental groups for cecal communities were observed in both 16S rRNA gene and metagenomic-assembled gene ordinations (Supplementary Figures S1, S2), suggesting that FMT administration specifically changes the community composition and functional profiles of the ceca but not the foregut. Based on this result, we focused on 16S rRNA gene and metagenomic comparative analyses of cecal communities to explore potential mechanisms through which early life exposure to FMTs leads to divergent adult bird phenotypes.

### Effect of FMT Donor Genetic Line on Cecal Microbial Structure and Function

**Figure 2:**
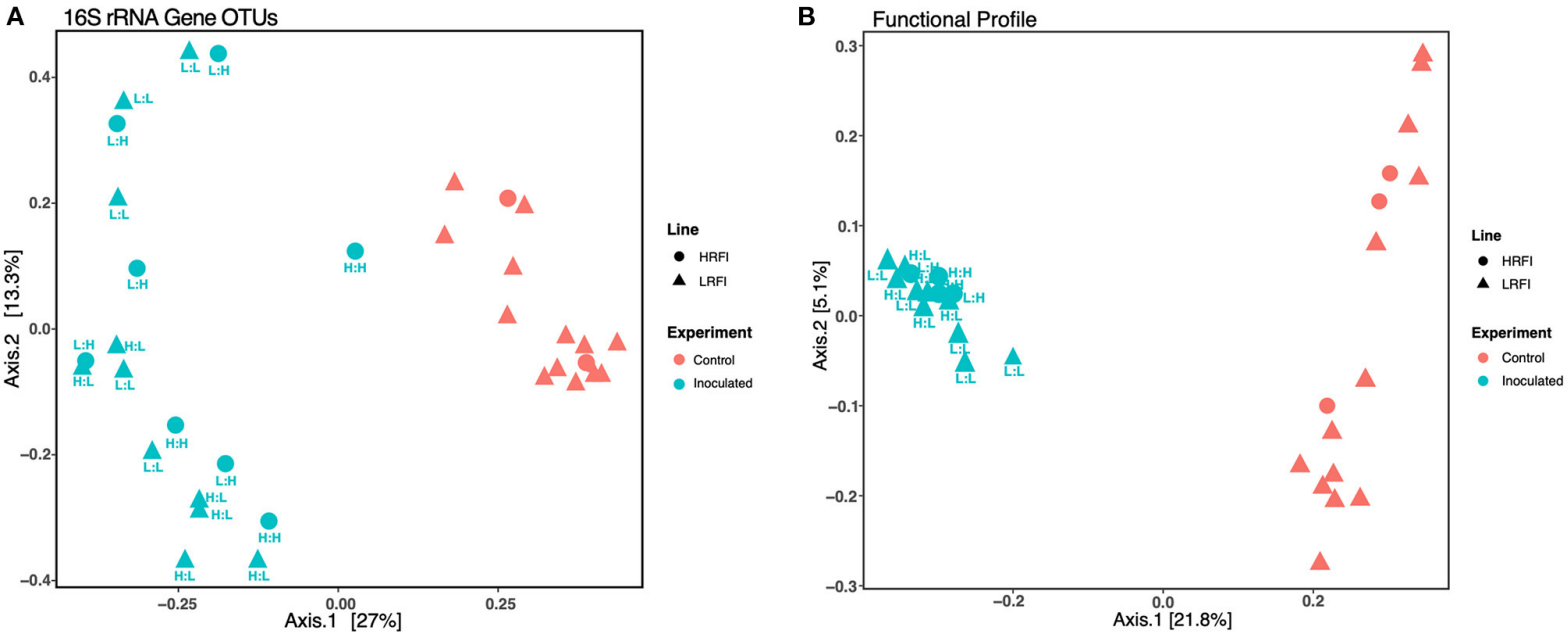
Cecal microbial community structure based on **(A)** 16S rRNA gene OTU frequencies and **(B)** metagenomic functional gene profiles. High and low RFI bird lines depicted as circles and triangles, respectively. Color depicts the inoculation state of the bird: coral = controls (not inoculated) and teal = inoculated. All inoculated samples are also labeled with the genetic line (L: low RFI and H: high RFI) of the FMT donor and recipient, respectively. For both 16 S rRNA-based microbial community structure and for functional profiles, differences between inoculated vs. uninoculated groups were significant (*p* < 0.01) as determined by Benjamini-Hochberg corrected PERMANOVA analysis as further described in the Supplemental Materials.

To test the relative effects of FMT administration and host genetics on cecal microbial community structure and functional profiles, we performed independent PERMANOVA analyses on each dataset (Supplementary Figure S2). As expected from the PCoA ordinations showing a large fraction of the variance explained by the FMT experimental group variable (Figure 2), FMT administration was the most significant factor in structuring community and predicted function (Benjamini-Hochberg corrected PERMANOVA *p* < 0.001, Supplementary Figure S1). The genetic line of the FMT donor also affected the community structure (p < 0.012) and functional profiles (*p* < 0.02), but only in low RFI birds receiving FMTs from high RFI donors (Supplementary Figure S1), suggesting that the genetic divergence of low and high RFI bird groups (22, 23) may also enrich certain GI microbial lineages that influence phenotype, although no significant differences were observed for community composition or functional profiles solely on the basis of genetic line for uninoculated birds (Supplementary Figure S1).

### FMT Enriched Lineages

**Figure 3:**
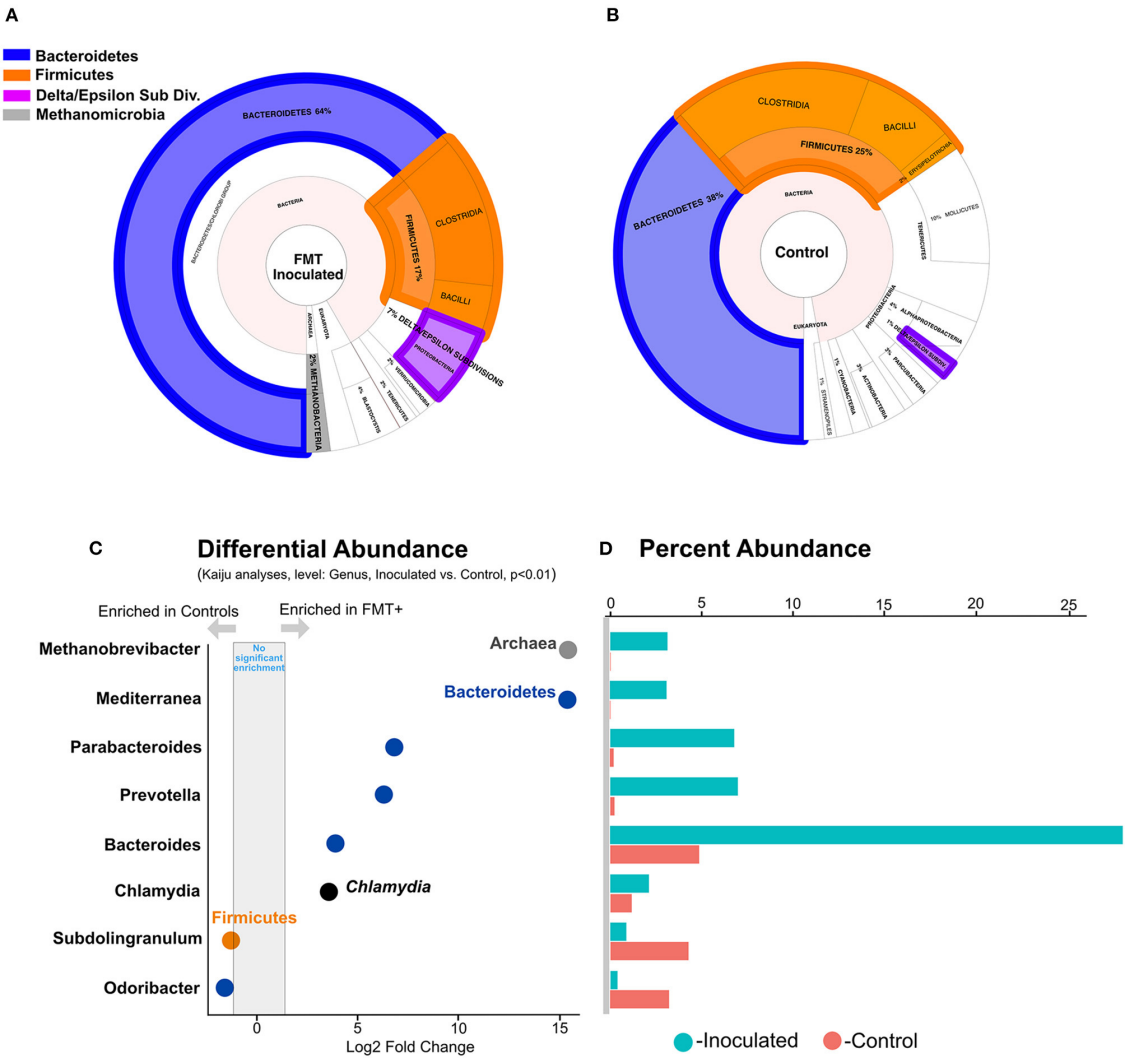
Microbial community composition and relative abundance of taxa with significantly different relative abundances between inoculated and uninoculated groups. **(A)** Phylogenetic assignment of all contigs assembled from FMT metagenomes. **(B)** Phylogenetic assignment of all contigs assembled from control metagenomes. **(C)** Differentially enriched lineages in controls and FMT recipients. **(D)** Percent abundance of each significantly differentially abundant lineage in FMT inoculated and uninoculated controls.

Next, to identify specific differentially represented cecal taxa, we used three approaches, phylogenetic assignment of metagenomic assembly contigs (Supplementary Figure S3A), metagenomic short reads (Supplementary Figure S3B), and 16S rRNA gene libraries (Supplementary Figure S3C) and showed that FMT administration resulted in Bacteroidetes enrichment over Firmicutes in the ceca at 6 weeks of age (Figure 3). We previously reported similar observations from two-week old birds administered a day-of-hatch FMT (17). Together, these reports imply that FMT-elicited cecal community modulation occurs rapidly (within 2 weeks) and persists up to typical market age of broiler chickens. FMT also elicited significant (*p* < 0.05) increases in the relative abundance of Epsilon- and Delta-proteobacteria (Supplementary Figure S4) and *Methanobrevibacter* spp., a hydrogenotrophic methanogenic genus (25, 26) (Figures 3A,B). Despite being a rare community member (~<2% average relative abundance), based on independent negative binomial (DESeq2) and linear (limma, ANCOM-BC) models implemented on our 16 S gene dataset (see materials and methods), *Methanobrevibacter* spp. was highly differentially enriched in the ceca of FMT recipients (Figures 3C,D, Supplementary Table S2). Despite the limited ability of any model to fully control type I errors and false discovery rates in compositional datasets (27), we note that FMT-driven *Methanobrevibacter* spp. enrichment is also independently corroborated by the obvious presence of Methanobacteria-assigned contigs generated exclusively from FMT metagenomes (Figures 3A,B, Supplementary Figure S3). The significant enrichment of *Methanobrevibacter* spp. and Deltaproteobacteria [e.g., *Desulfovibrio* spp., a genus representing potential syntropic partners (19)] highlights the ecological importance of archaea in cecal fermentation energetics and suggests that syntrophic interactions between archaeal and bacterial partners may modulate the composition, activity, and efficiency of the highly abundant cecal fermentative community (18), influencing feed efficiency (28).

Next, using quality criteria (80% completeness and 10% redundancy cut-offs) suggested elsewhere (29), we assembled one Archaeal and seventeen Bacterial high-quality MAGs from our cecal co-assemblies (see Supplementary Table S1). More specifically, one Archaeal MAG, classified as a *Methanobrevibacter* spp., and seven Bacterial MAGs (six classified as members of the Bacteroidetes phylum and one identified as a Deltaproteobacterial lineage) were recovered from FMT inoculated co-assemblies while the control co-assemblies yielded ten Bacterial MAGs (three assigned to the domain Bacteria, five assigned to the Bacteroidetes phylum, and two assigned as Firmicutes). As a survey of the chicken cecal genomic landscape, we produced a bacterial phylogenomic tree that includes 469 previously reported cecal MAGs (29), 91 genomes generated from cecal lab culture isolates, and our bacterial MAGs (Supplementary Figure S7) using a 92 bacterial core gene reconstruction model (30). This survey shows that our bacterial cecal MAGs and lab isolates mostly represent novel clades relative to a previous survey (29), expanding the known phylogenomic diversity of cecal communities. Our single Archaeal MAG (MAG_Arch1), underwent a separate focused analysis, as described below.

### Cecal Metabolic Activity and Genetic Regulatory Pathway Predictions

**Figure 4:**
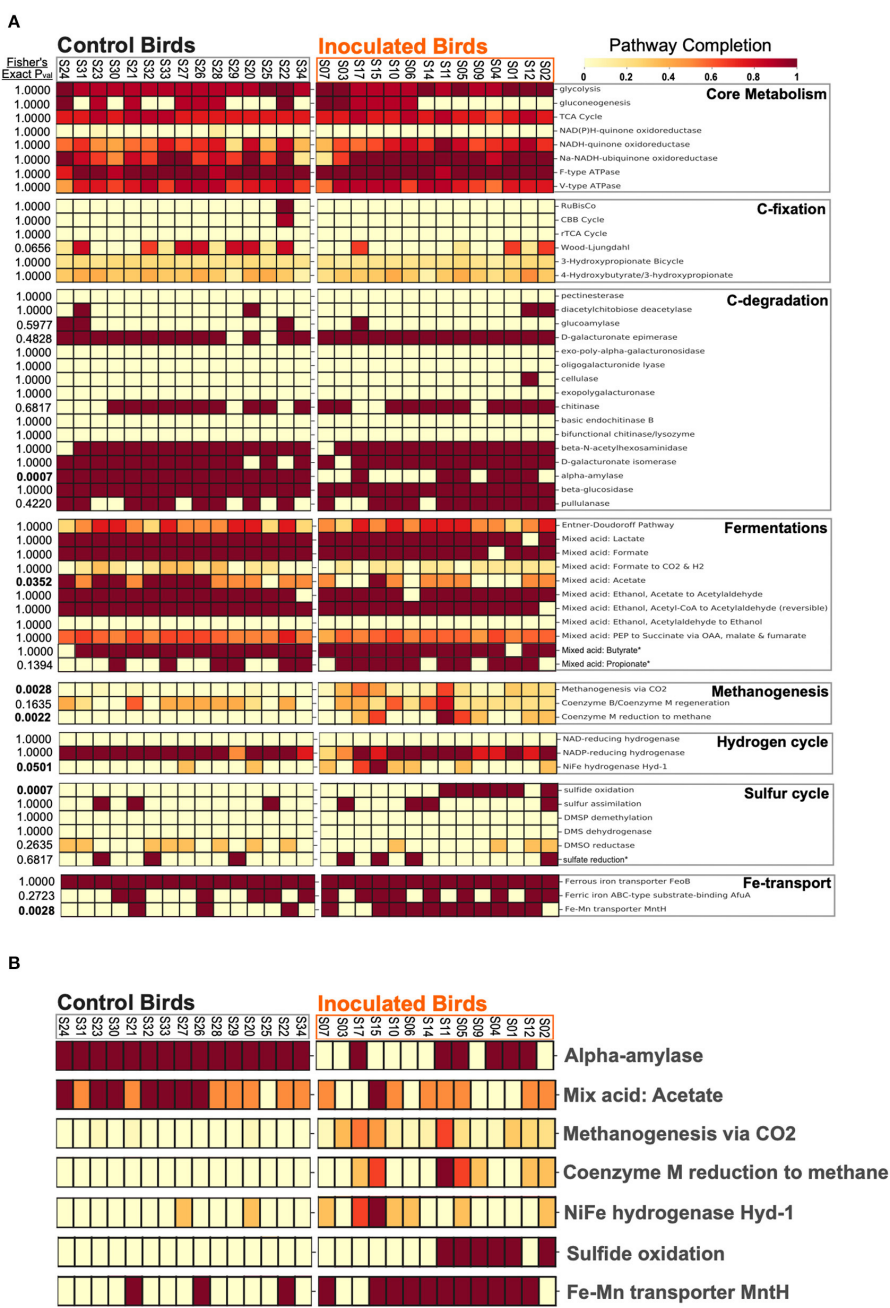
Predicted completion of KEGG metabolism modules for **(A)** all cecal metagenomic assemblies. **(B)** Predicted metabolism modules from **(A)** with significant (student *t*-test, *p*-value < 0.01) differences between FMT inoculated birds and controls.

Significant (*p* < 0.05) differences in metabolic pathway predictions between our treatment groups were observed for methanogenesis, Fe-Mn transport, and hydrogen- and sulfur- cycling (Figure 4). The larger pathway categories of core metabolism, carbon fixation, carbon degradation, and fermentation were not significantly different between experimental groups (Figure 4A).

Fermentation products, predominantly SCFAs, can be viewed as the symbiotic currency between cecal microbiota and the avian host (12). We show that, despite changes in the microbial community structure elicited by the FMT, the genetic potential for polysaccharide degradation and fermentations appears to be conserved at the pathway level between experimental groups. This was unexpected since enrichment of highly abundant Bacteroidetes lineages in FMT samples (Figures 3C,D) suggests that carbon degradation and/or fermentation-related activities, metabolic hallmarks of the Bacteroidetes (31), may differentiate experimental groups. To explore this further, we explicitly tested for enrichment in genes involved in the final step in the production of butyrate, a major GI microbiome terminal metabolite crucial to host intestinal health (32), from butyryl-CoA. The butyryl-CoA: acetate CoA-transferase pathway or butyryl-CoA phosphorylation and final transformation by butyrate kinase are the major penultimate steps leading to the production of butyrate in fermentative communities (33). We report that the butyryl-CoA: acetate CoA-transferase pathway was enriched in the ceca of FMT birds (Supplementary Figure S5), suggesting that subtle differences in fermentation efficiencies may indeed be elicited by FMT administration and may go unnoticed when examining the experimental groups at the fermentative pathway-level. Butyryl-CoA: acetate CoA-transferase butyrate production is reported to increase acetate uptake and stoichiometric proton export driving net ATP generation via oxidative phosphorylation at mildly acidic pH (34, 35). Thus, FMT administration appears to modulate the production of major GI microbiome fermentative, symbiosis-linked, products such as butyrate.

Interestingly, some insertion sequences (ISs), the smallest and most common autonomous transposable elements (36), phylogenetically assigned to the Bacteroidaceae were also differentially enriched in the FMT samples relative to controls (Supplementary Figures S6A,B). Drastic expansion in IS elements loads are generally associated with genome rearrangement and reduction (36). Our results thus suggest a degree of on-going genome specialization, a result of symbiotic associations in cecal communities of FMT recipients.

### Communal Hydrogenotrophy: A Mechanism of Cecal Fermentative Optimization?

**Figure 5:**
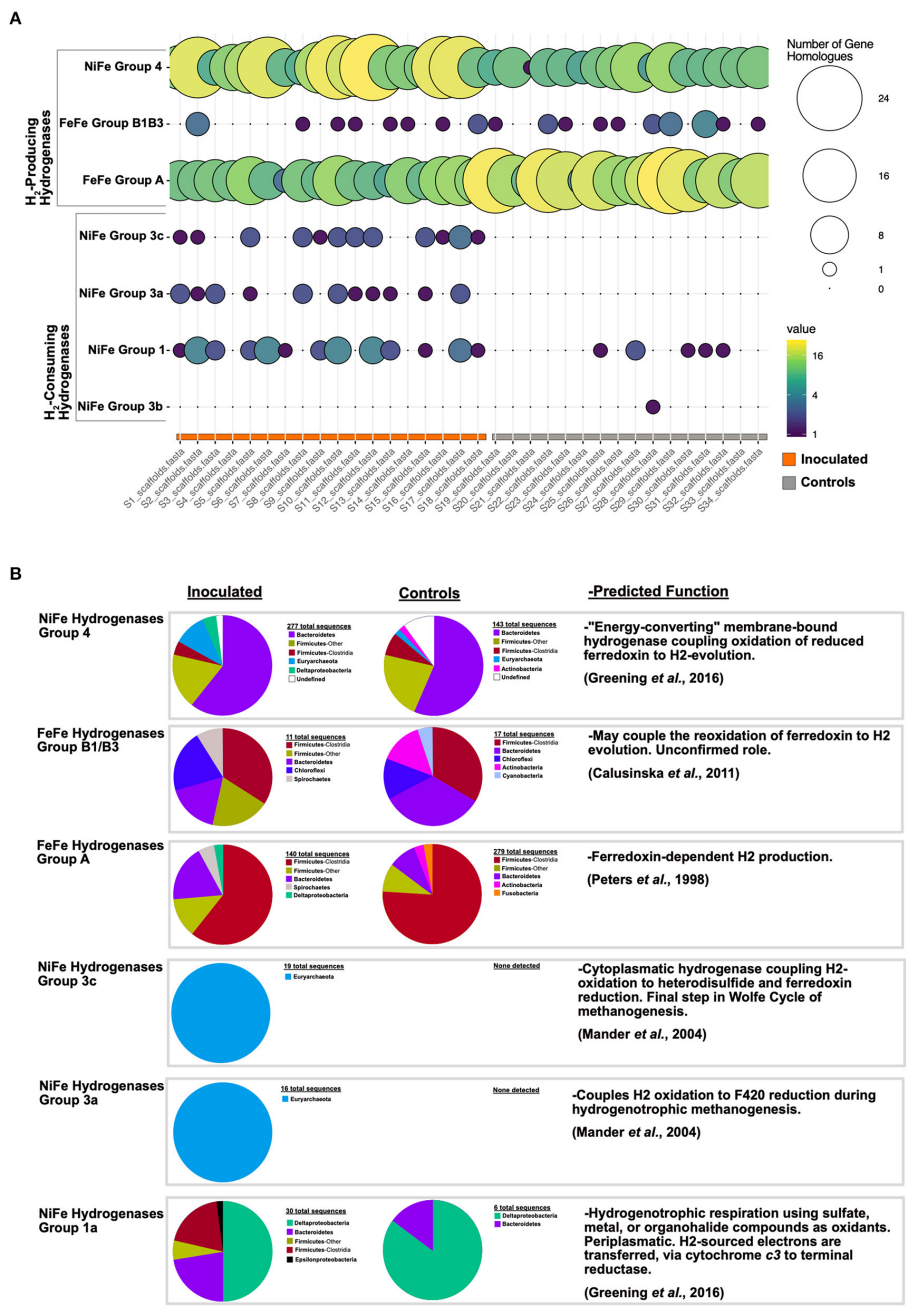
Hydrogenase gene survey. **(A)** pHMM-based hydrogenase survey of all cecal metagenomes. Circle size and color reflect the number of detected gene homologs per hydrogenase category. **(B)** Phylogenetic assignment and predicted function for hydrogenase gene homolog categories from FMT inoculated and control cecal metagenomes.

We exclusively detected in the cecal communities of FMT recipients a methanogenic archaeal lineage (*Methanobrevibacter* sp.), sulfate reducing bacteria (SRBs; known archaeal syntrophic partners such as *Desulfovibrio* spp.), and *mcr*A genes, markers for archaeal methanogenesis (Figures 3, 4, Supplementary Figures S4, S7). Methanogen gene markers and methane production have been previously detected in chicken ceca and feces, respectively (37, 38). Additionally, there is a report of lower methane production and lower feed efficiency in goslings following a caecectomy (39). Building upon these important previous results, our work is the first, to our knowledge, to explicitly consider the thermodynamic role played by archaeal methanogens in the poultry cecum associated with significant effects on phenotype. Methanogenic archaea compete with SRBs for common substrates such as acetate and, in fermentative environments, H_2_, concomitantly generating sulfide and methane (18). Sulfate reduction by SRBs and microbial catalysis of sulfur-containing amino acids in proteins, including methionine, cysteine, homocysteine, and taurine, as reported from humans (40) and rat ceca (41), potentially render the avian ceca a net sulfidic environment. In our data, Epsilonproteobacteria and the potential for sulfide oxidation, a characteristic activity of this lineage, were exclusively detected in the FMT group (Figures 3, 4, Supplementary Figure S4). Intriguingly, sulfide, thiosulfate, and elemental sulfur oxidation by Epsilon-proteobacteria must be coupled to the reduction of oxygen or nitrate (42). Interestingly, hydrogenotrophic growth with elemental sulfur reduction, a likely redox couple in the cecal environment, has been reported for Epsilonproteobacterial isolates from seafloor hydrothermal habitats (42). This suggests the possibility that cecal Epsilonproteobacteria are predominantly hydrogen oxidizers in the ceca. In FMT birds, the concomitant enrichment of archaeal methanogens, hydrogenotrophic Delta- and Epsilon-proteobacteria, and the increased prevalence of NiFe hydrogenases specific to these lineages (Figure 5), strongly suggest hydrogen cycling as a key ecological process in FMT-linked cecal communities that may influence the emergent phenotypes of the host, as further discussed below.

### Cecal Hydrogen Cycling and Fermentative Modulation

SCFA production rates and profiles depend on the partial pressure of H_2_ resulting from its production and consumption by hydrogenases of fermenters and hydrogenotrophs, respectively (43, 44). The hydrogenase profiles in our experiment were dominated by NiFe (Group 4) and FeFe (Groups A and B1B3) hydrogenases, involved in H_2_ generation from energy conversion and H_2_ splitting coupled to electron shuttle reduction (45, 46), respectively (Figure 5A). Based on taxonomic assignments of hydrogenase genes (Figure 5B) NiFe Group 4 and FeFe Group A and B1B3 hydrogenases, lineages that may generate H_2_ in the ceca of FMT-inoculated birds include Bacteroidetes, Firmicutes, Deltaproteobacteria, and, to a lesser extent, Chloroflexi and Spirochetes. Of the four other hydrogenase groups detected in this survey, two were exclusively observed in FMT-inoculated communities, and are both H_2_-consuming hydrogenases (Figure 5A). Archaeal Mn-reducing cytoplasmatic NiFe hydrogenases (Groups 3a and 3c), involved in hydrogenotrophic methanogenesis (47), were only observed in the cecal communities of FMT-inoculated birds (Figure 5). Similarly, oxygen-tolerant cytoplasmic Group 1 NiFe hydrogenases, involved in hydrogenotrophic respiration coupled to sulfate, metals, and other oxidants (48), were enriched in FMT communities. Interestingly, a Group 1 hydrogenase sequence, detected exclusively in FMT-inoculated communities, is of Epsilonproteobacterial provenance (Figure 5B). This observation supports a potential hydrogenotrophic role (coupled with elemental sulfur reduction) for some Epsilonproteobacteria in the cecal communities of FMT-inoculated birds. Overall, our results suggest that net H_2_ gas consumption by hydrogenotrophic organisms promotes additional hydrogen formation, electron carrier replenishment, and continued fermentation (44). We note that FMT-elicited microbial hydrogen cycling may provide a thermodynamic advantage that, over a commercial chicken's lifetime (~6 weeks), could explain significant increases in adult bird weight and feed conversion efficiency relative to controls. These results, to our knowledge, document the first described mechanism linking FMT inoculation, cecal hydrogen cycling, and emergent avian host phenotypes.

### Avian-Microbe Hydrogen Mutualism: Archaeal Streamed-Lined Genomes and Evolutionary History

**Figure 6:**
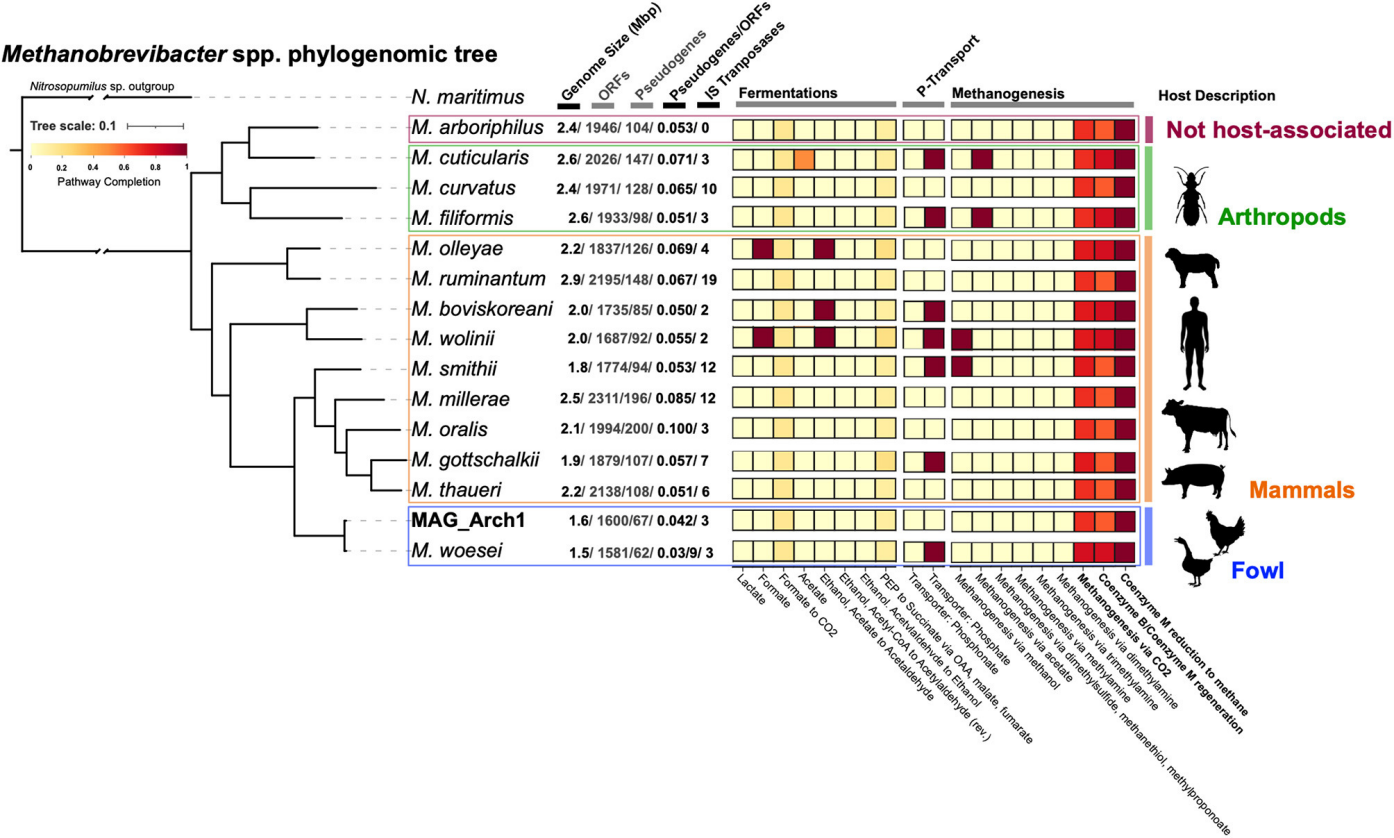
Phylogenomic tree depicting the closest phylogenetic relatives of MAG Arch1. Side panel depicts percent completion prediction for various metabolism modules associated with fermentative and methanogenic activity. The schematics to the right depict the animal host source of each genome in the tree.

Despite the low abundance of Archaea in the ceca, Methanobrevibacter *spp*. are the most differentially enriched lineage in the FMT-inoculated communities (Figure 3, Supplementary Table S2). To better understand their genomic context, a near-complete metagenomic-assembled genome (MAG; MAG_Arch1) was recovered from FMT-inoculated samples and phylogenomically compared to 15 publicly available, host-associated, *Methanobrevibacter* genomes (Figure 6). MAG_Arch1 is most closely related to a *Methanobrevibacter woesei*, a strict hydrogenotrophic methanogen isolated from goose fecal enrichments (25), and more distantly related to non-fowl hosted members of this genus (Figure 6). An *mcrA* gene sequence assigned to the Methanobacteriales order, (of which *Methanobrevibacter* is a member), with 100% similarity to the *mcrA* of MAG_Arch1, was also independently detected from FMT metagenomic assemblies (Supplementary Figure S6). Collectively, our metagenomic observations corroborate 16S rRNA gene survey reports of methanogenic lineages (37) and active methane production (38) in cecal material collected from 56 to 72 week-old layer chickens. *Methanobrevibacter* genomes recovered from fowl are significantly smaller than those recovered from non-fowl hosts (Figure 6). Their genomes (1.6Mbp in size, encoding 1,600 proteins for MAG_Arch1) are comparable to that of *Methanothermus fervidus* V24S^T^ (1.24 Mbp encoding 1,300 proteins), one of the smallest known free-living Archaea (49). This highly streamlined genomic architecture, in addition to low ORF-normalized pseudogene loads and high gene coding densities (Figure 6), as reviewed elsewhere (50, 51), strongly suggests that these fowl-associated Archaea are highly specialized cecal symbionts.

### Methanobrevibacter and Performance-Related Outcomes in Vertebrates: An “Ancestral Link”?

Well-preserved dinosaur fossils date the anatomical and physiological emergence of the avian GI tract to the Early Cretaceous (52). Thus, ancient and modern ceca are fermentative hotspots and redox-optimal niches for hydrogenotrophic methanogenesis that represent a 160-million-year-old history of microbe-avian mutualism. The presence of *Methanobrevibacter* spp. has been correlated with performance-related outcomes in avian (this study), mammalian (53–55), and reptilian (56) hosts. Methane is a potential therapeutic gas capable of suppressing inflammation, oxidative stress, and apoptosis in inflammatory bowel disease animal models (57). Methane generation has also been recently proposed as a biologically universal adaptive stress response (58), highlighting the central role that methane cycling may play in microbe-animal mutualism.

Various aspects of modern poultry husbandry (e.g., biosecurity protocols and single age cohorts) constrain microbial inoculation of commercial poultry relative to wild populations. Notably, after a single generation in captivity, grouse have been reported to have significantly less diverse microbial communities and atrophied cecal micromorphology relative to non-captive controls (59). Also, significant differences in the GI microbiome have been shown between feral and domestic chickens. For example, microbial alpha diversity of the cecal microbiota was higher for commercial than for feral chickens and microbial metabolic pathways for L-proline biosynthesis were enriched in the feral group (60). Domestication (effective captivity) in the case of chickens, changes the ecological context, particularly microbial exposure (61) at different life stages that may result in divergent trajectories of microbial succession. We posit that FMT administration in our experiment mimics ancestral environmental microbial exposure (e.g., early contact with methanogens, which to our knowledge are not vertically transmitted) and results in an optimized microbial ecological function in the chicken ceca. In a natural setting, where chicks are brooded under the wings of their mothers, early-life exposure may also occur via coprophagy (62). Optimal cecal microbial community establishment and succession (e.g., the promotion of specific FMT-delivered lineages serving as cecal hydrogen sinks) may result in enhanced NSP digestion, fermentation, and SCFA production that can modulate the avian host phenotype.

### Ecology of Red Jungle Fowl: Implications for Modern Poultry Rearing

Further supporting our “ancestral link” hypothesis, field reports of wild Red Jungle Fowl (RJF, *Gallus gallus*) from the Kanchanaburi province in Thailand, one of the earliest sites of RJF domestication, make the following observations: i) RJF are exclusively observed in or around bamboo forests, ii) newly hatched, downy-coated chicks eat termites as their primary food source, and iii) adult bird diets are omnivorous, but primarily feed on plant materials (various fruits and bamboo, rice, bean, and grass seeds) and secondarily, insects (including termites, ants, beetles) (63). This report ecologically contextualizes the main findings of our study as follows: Newly hatched chicks in the ancestral environment are continuously exposed to methanogenic assemblies, specifically, *Methanobrevibacter* spp., and their syntrophic partners (e.g., *Desulfovibrio* spp.) that abound in the hindguts of bamboo infesting termites (64). This early-life exposure to methanogenic communities, through chick termite foraging, likely primes the cecal microbiota for efficient hydrogen cycling and optimal fermentation of plant-sourced materials, the primary diet of adult birds.

The ecology of RJF, the wild stock of all domesticated chickens (*Gallus gallus domesticus*), reinterpreted through our phenotype-linked metagenomic results provides important insights regarding the effects of direct-fed microbials on poultry. In nature, optimization of the adult GI microbiome for a plant-derived carbohydrate rich diet begins with continuous exposure to complex methanogenic communities, playing a role in hydrogen cycling and fermentative output, through the termite rich diet of young chicks. Our early-life FMT exposure experiment mimics the ecological microbial exposure pattern of wild RJF and suggests that minimal early-life modification in commercial chicken rearing may be able to optimize cecal fermentative activities, resulting in the enhancement of agriculturally desirable bird phenotypes. Such approaches could provide valuable alternatives to the use of antibiotics as growth promoters.

## Conclusions

Methanogens and sulfur-cycling Proteobacteria were enriched in the cecal communities of performance-enhanced FMT inoculated birds and likely function as hydrogen sinks that serve the collective thermodynamic needs of primary fermenters. We propose that enhanced potential for hydrogen disposal in the cecum, elicited by FMT transplantation, increases fermentation efficiency. This provides a direct advantage to the bird by allowing the catabolic use of otherwise indigestible fibers and provides a plausible microbial mechanism through which FMT administration results in enhanced, and agriculturally desirable, bird phenotypes. These results may suggest novel categories of pre- and probiotics that can enhance antibiotic-free poultry production. Our work explicitly considers the GI microbiome as a basic element of the production system of food animals and provides a new evidence-based paradigm in food animal husbandry, grounded in eco-genomics, offering new frontiers for sustainable agriculture.

## Materials and Methods

### Experimental Design

The design of the inoculated group was a factorial design crossing genetic line between donors and recipients of fecal microbiome transplants (FMTs) administered once to newly hatched chicks (see Supplementary Figure S2 for a graphical summary). Genetic line for each bird (Arkansas Randombred) was classified as High Relative Feed Index (HRFI) or Low Relative Feed Index (LRFI), referring to low or high feed efficiency, respectively [Note: low RFI means lower amount of feed consumed than expected (i.e., higher efficiency) and high RFI means higher amount of feed consumed than expected (i.e., lower efficiency)]. In total, there were 40 inoculated chicks (20 female, 20 male) and 63 uninoculated chicks (35 female, 28 male) as controls. Of these, 34 birds were used for 16 S rRNA gene surveys (15 uninoculated and 19 inoculated) of jejunum, ileum, and cecum contents. Thirty of the same birds (16 uninoculated and 14 inoculated) were used for metagenomic sequencing of cecal contents only. See Supplementary File 1 for full details.

To prepare the inoculum, GI tracts from donors were collected via necropsy at 6 weeks of age. Immediately following necropsy, intact ceca were transferred to an anaerobic chamber where cecal contents and mucosal scrapings of the cecal walls were collected. Cecal inocula were slowly frozen to −20 deg C and then stored at −80 deg C until use. Immediately prior to inoculation, donor material was thawed and mixed in a 1:3 ratio (w:v) with sterile PBS and 0.2 mL of this suspension was administered once to day-of-hatch chicks via oral gavage with a 1 mL syringe and gavage needle, as previously described (17). Following inoculation, chicks were reared in standard floor pens, segregated by treatment group. Standard feed formulated by the UGA feed mill was provided *ad libitum* with starter feed (23.0% crude protein, 6.0% crude fat, 2.5% crude fiber, 1.0% calcium, 0.48% available phosphorus; ca. 3,100 kcal/kg) for the first 2 weeks, and subsequently switched to grower feed (21.0%, crude protein, 6.5% crude fat, 2.4% crude fiber, 1.0% calcium, 0.45% available phosphorus; ca. 3,200 kcal/kg). At 6 weeks of age, inoculated and control birds were humanely euthanized by cervical dislocation after electrical stunning and the jejunum, ileum, and ceca removed via necropsy.

Standard measures of bird performance were collected through the experiment. These included weekly body weight gain, feed consumption, and relative feed index (RFI).

### DNA Extraction and Sequencing

Approximately 0.25 mg of intestinal contents were used for DNA extraction with the MoBio (Carlsbad, CA) Power Soil DNA extraction kit following manufacturers recommendations. PCR using the primers 519F (5′-CAGCMGCCGCGGTAATWC-3′) and 926R (5′-CCGTCAATTCCTTTRAGGTT-3′) was conducted as previously described (9, 65, 66) with a barcoding scheme detailed elsewhere (67). Amplicons were normalized with the Invitrogen SequalPrep kit (Carlsbad, CA) and sequenced on the Illumina MiSeq using the 2 × 250 bp v2 kit.

### Sequence Quality, Trimming, and Assembly

Metagenomic and 16 S rRNA targeted paired-end Illumina libraries were inspected for quality parameters and repetitive sequences using the FastQC software package (https://www.bioinformatics.babraham.ac.uk/projects/fastqc/). Adapter trimming and low-quality sequence removal was performed using the tool Trimmomatic version 0.39 (68) using the following parameters: -phred33, Illumina adapter removal, min length:36, sliding window 4:15. A *de novo* metagenomic co-assemblies for individual bird metagenomes, separate inoculated and controls, and all metagenomes were performed using merged forward and reversed adapter trimmed sequences with metaSPAdes v.3.14.1 (69) and subsequently analyzed as described below.

### Metagenomic Assembled Genomes

Metagenomic assembled genomes (MAGs) were binned from independent FMT inoculated and non-inoculated assemblies using Metawrap (70) under default binning parameters. The resulting MAGs were optimally consolidated using DASTool (71) and visually inspected using Anvi'o v5 (72). Completeness and redundancy of each of the final bins was assessed using CheckM (73).

### 16S rRNA Gene Clustering, Taxonomic Assignment, and Differential Enrichment Testing

Operational Taxonomic Units were generated from high quality 16 S rRNA gene sequences using the Usearch pipeline (74) and representative sequences were classified against SILVA release 138 (75). The following R packages, which include both negative binomial and linear regression models, were used for exploring experimentally-driven differential enrichment of microbial taxa: *DeSeq2* (rlog transformed per-sample shrinkage of fold-change) (76), *limma* (TMM normalized and empirically Bayes smoothed Limma-Voom analysis) (77), and ANCOM-BC (78).

### Metagenomic Short Read Taxonomy, ORF Calling, Annotation, and Gene-Targeted Analyses

Taxonomic and functional analyses of unassembled metagenomic reads were performed using the Kaiju pipeline for taxonomy (79) against the NCBI non-redundant database. Open reading frame (ORF) identification and, subsequently, prokaryote predicted protein product annotations were performed with Prodigal v2.6.1 (80) implemented in Prokka (81). All ORFs were also aligned against the NCBI non-redundant (nr) database (accessed in November 69) using BLASTp (82) for closest homolog taxonomy and functional annotation supplementation. Targeted single gene homolog searches were performed using BLASTp 2.2.30+ (*E*-value threshold = 1E−30, min. identity = 50%) against predicted protein sequences inferred from unbinned metagenomic contigs. Lastly, we developed a python executable tool implementing libraries of profile Hidden Markov Models (pHMMs) representing microbial catabolism genes called *LithoGenie* (https://github.com/Arkadiy-Garber/LithoGenie) and used it to search for hydrogenase gene homologs across metagenomes. Additional information on *LithoGenie* development and implementation is found in the Supplementary Materials and methods section. Taxonomic assignment of predicted hydrogenases was done based on closest hits to the NCBI nr protein database using BLASTp and standard algorithm parameters. To assess coverage as a proxy for gene abundance, quality-trimmed short metagenomic reads were mapped to our *de novo* assembled genes using Bowtie2 (83). Results were concatenated into read count tables for downstream statistical analyses using the R package vegan (84) implemented in phyloseq (85). Ordination visualization was performed as Principal Coordinate Analysis (PCoA) on Bray-Curtis distances for 16S rRNA genes frequencies and total ORFs independently. Differences between inoculated and uninoculated groups were formally tested using a Benjamini-Hochberg corrected PERMANOVA analysis and a significance cut-off of *p* < 0.01.

### Pathway Completion Estimates

Predicted protein products were annotated against the KEGG database (86) via GhostKOALA (87) with the following parameters: taxonomy group, Prokaryotes; database, genus_prokaryotes; accessed February 2021. The output annotation file was used for pathway completion assessment and visualization using KEGG-decoder.py (88). Paired Student's *t*-test was used for testing significant differences (*p* <0.05) in mean completion estimates for all metabolic pathway categories.

### *Methanobrevibacter spp*. Comparative Genomic Analysis

The archaeal phylogenomic tree was generated using the GToTree package (89). Briefly, our MAG and other related public genomes classified as *Methanobrevibacter* spp. were ran against a GToTree's “Archaea” HMM collection of domain-specific single copy genes resulting in a Muscle (90) concatenated protein alignment subsequently trimmed with TrimAl (91). FastTree2 (92) was used for tree construction and visualizations were performed on FigTree (https://github.com/rambaut/figtree). IS transposase family sequences were identified as previously described for metagenomic annotation and pseudogene detection was performed using *pseudofinder* (93).

## Data Availability Statement

The datasets presented in this study can be found in online repositories at: https://www.ncbi.nlm.nih.gov/bioproject/?term=PRJNA812961 and https://gold.jgi.doe.gov/study?id=Gs0144355.

## Ethics Statement

The animal study was reviewed and approved by the USDA National Poultry Research Center Animal Care and Use Committee.

## Author Contributions

GR led bioinformatics analyses, led interpretation of results, and wrote the manuscript. JK, IV, and AG contributed bioinformatics analyses and interpreted results. MB and FG-C contributed to study design and performed experiments. NC contributed to study design, performed experiments, designed bird genetic study, and interpreted results. BO led experimental design, interpreted results, and advised GAR. All authors contributed to the article and approved the submitted version.

## Funding

Funding was provided by USDA NIFA grants 1015210 and 1011327 and research award F053 from the US Poultry and Egg Foundation.

## Conflict of Interest

The authors declare that the research was conducted in the absence of any commercial or financial relationships that could be construed as a potential conflict of interest.

## Publisher's Note

All claims expressed in this article are solely those of the authors and do not necessarily represent those of their affiliated organizations, or those of the publisher, the editors and the reviewers. Any product that may be evaluated in this article, or claim that may be made by its manufacturer, is not guaranteed or endorsed by the publisher.
