# Supplementary material for: Cecal Microbial Hydrogen Cycling Potential Is Linked to Feed Efficiency Phenotypes in Chickens
Source: Front Vet Sci. 2022 Jun 21;9:904698. doi: 10.3389/fvets.2022.904698 (PMC9255636; doi:10.3389/fvets.2022.904698)
Supplement: Supplementary file 1 [file Table_1.DOCX]

*Supplemental Information for Frontiers in Veterinary Science*

**Cecal microbial hydrogen cycling potential is linked to feed efficiency phenotypes in chickens**

Gustavo A. Ramírez^1,2^, Jitendra Keshri^1^, Isabella Vahrson^1^, Arkadiy I. Garber^3^, Mark E. Berrang^4^, Nelson A. Cox^4^, Fernando González-Cerón^5,6^, Samuel E. Aggrey^6^, Brian B. Oakley^1*^

Author Affiliations:

1: College of Veterinary Medicine, Western University of Health Sciences, Pomona CA, USA.

2: Leon H. Charney School of Marine Sciences, Haifa University, Haifa, Israel.

3: School of Life Sciences, Arizona State University, Tempe AZ, USA.

4: Poultry Microbiological Safety and Processing Research Unit, USDA Agricultural Research Service, Athens, GA, USA.

5: Departamento de Zootecnia, Universidad Autónoma, Chapingo, Estado de México, México.

6: NutriGenomics Laboratory, Department of Poultry Science, University of Georgia, Athens, GA, USA.

*Corresponding Author:

Brian B. Oakley

[boakley@westernu.edu](mailto:ramirezg@westernu.edu)

Western University of Health Sciences

College of Veterinary Medicine

Pomona, CA 91766

Figure S1. PCoA ordination plot based on ASV frequency table Bray-Curtis dissimilarity distance. All samples depicted here had at least 500 sequence counts. Red and yellow circles represent inoculated and uninoculated cecal samples, respectively. Foregut samples are depicted as follows: green and cyan circles represent inoculated and uninoculated Ileum communities, respectively, and blue and purple circles represent inoculated and uninoculated Jejunum communities.

Figure S2. PERMANOVA analysis of Bray-Curtis distances for bird genetic line and FMT administration as explanatory variables was implemented in the R package *vegan* using the *adonis* function on A) 16S rRNA gene data and B) functional profile distances.

Figure S3. A. Krona summarized metagenomic contig predicted community composition. B. Kaiju short read predicted community composition. C. Kaiju 16S rRNA ASV predicted community composition. D. Kaiju summarized metagenomic contig predicted community composition


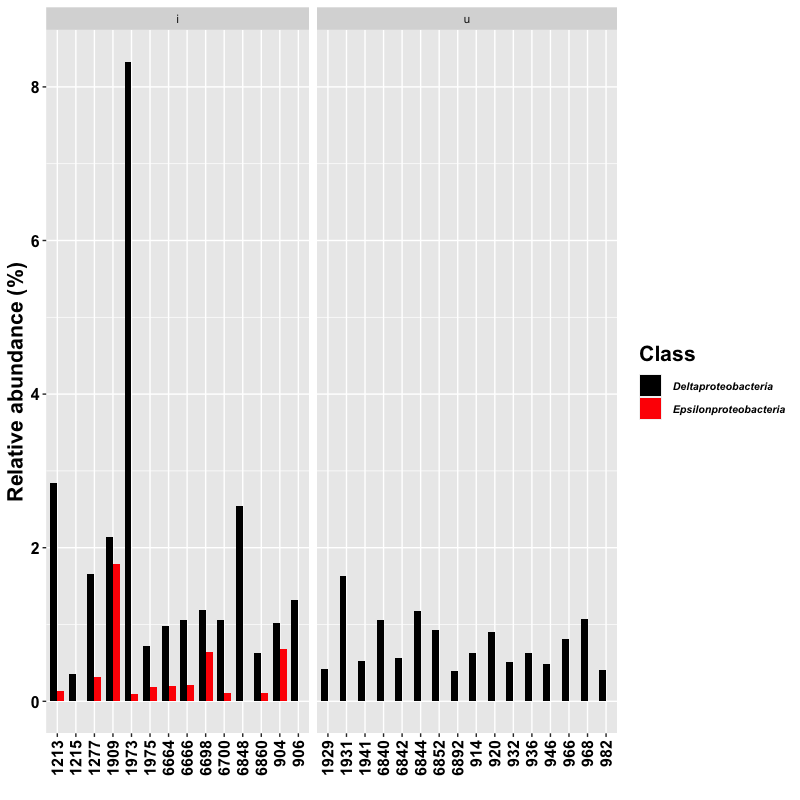


Figure S4. Relative community abundance of Deltaproteobacteria and Epsilon proteobacteria ASVs in (I) inoculated and (U) uninoculated samples.

Figure S5. Gene coverage for butyrate pathway markers.

Figure S6.

*Insertion sequence elements and gene expression regulation*

Most IS elements were assembled in sort contigs containing less than 5 protein sequences often annotated as hypothetical products (data not shown). Fortunately, a single long coting (23K bp) contained an IS 1380 family IS612 transposase gene, an IS element differentially enriched in FMT-elicited communities (Figure 3B & F). This IS 1380 family IS612 transposase is flanked by an efflux ABC-transporting ATP-binding protein, an ABC2-type transport permease, dual integrases, a putative transposon excisionase T916, and various hypotheticals (Figure 3F). Interestingly, gene up-regulation mediated by promoter integration or hybridization upstream from otherwise inactive genes is another possible consequence of IS element integration (Siguier et al. 2014). Depending on the target site on the genome, these mechanisms affect host cell physiology and have been experimentally shown to increase resistance to antimicrobials by affecting efflux pump activity in model organisms (Olliver et al., 2005, Jellen-Ritter & Kern, 2001). The insertion of the IS 1380 family IS612 transposase directly in between an integrase and ABC transporter efflux pump, in addition to the presence of a putative T916 transposon excisionase in the genomic vicinity (Roberts & Mullany, 2011), strongly suggests that day-of -hatch FMT administration confers enhanced antimicrobial resistance in the cecal communities of adult birds. A direct link between the FMT-conferred potential for enhanced antimicrobial resistance of cecal communities and the measured enhanced growth phenotype of the bird host remains elusive.


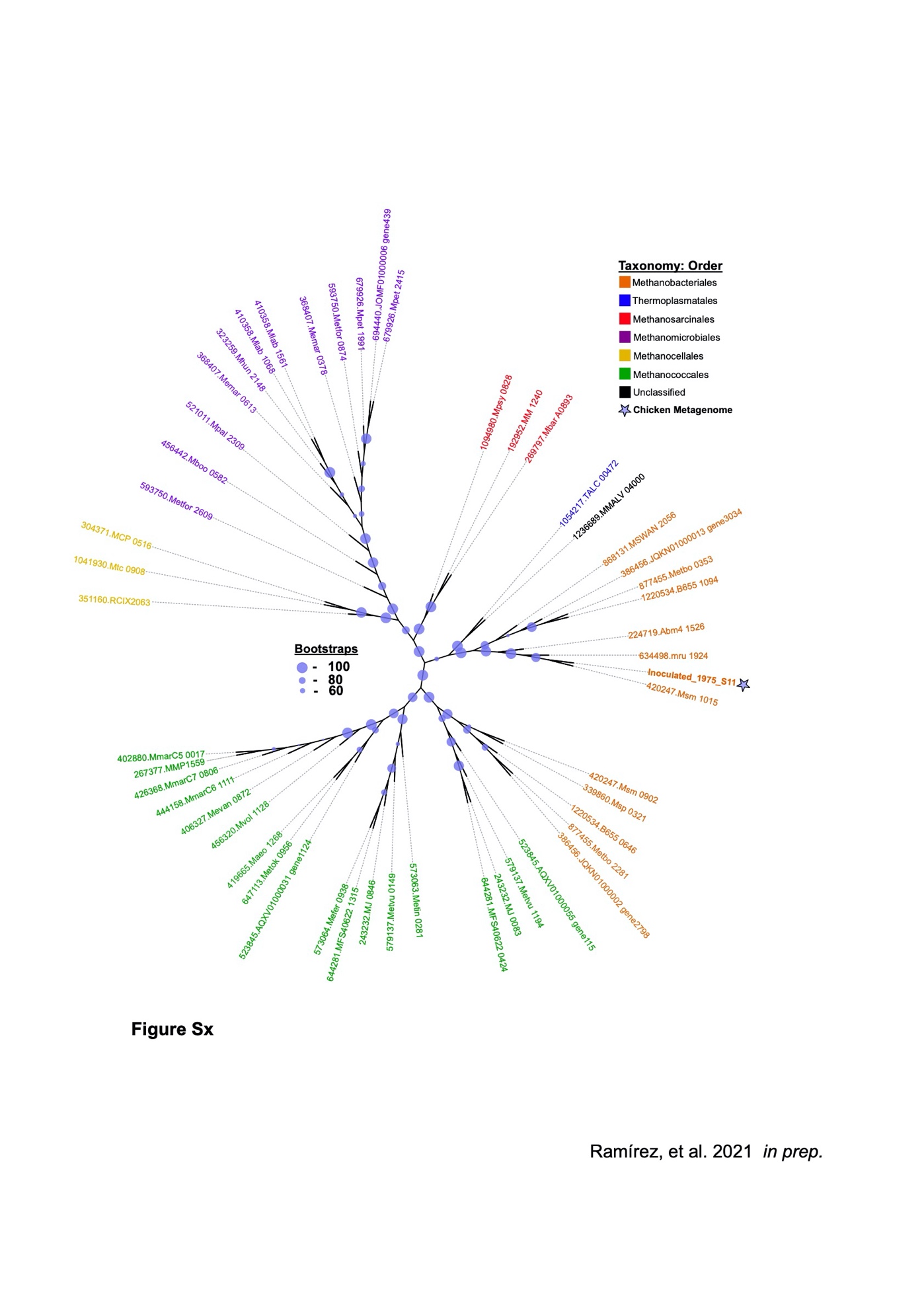


Figure S7. McrA gene phylogenetic tree. McrA gene taxonomic provenance is color coded. The single McrA gene variant recovered in this study is predicted as belonging to the Methanobacteriales and is depicted with a star.

Figure S8. Phylogenomic meta-analyses of cecal bacterial genomes. This UBCG tree summary, generated using a 92 conserved gene pHMMs, contains MAGs from this study (red and blue, see Table S1 below), metagenome assembled genomes from other studies (orange, Glendinning et al. 2020), and cecal cultured isolates from our own lab (green, Gold study ID Gs0144355).

| **assembly_id** | **label** | **taxid** | **num_SCG_hits** | **uniq_SCG_hits** | **perc_comp** | **perc_redund** | **num_SCG_hits** | | **in_final_tree** |
| --- | --- | --- | --- | --- | --- | --- | --- | --- | --- |
| FMT_control_Bacteria_bin.10 | Uninoc_bin10 | NA | 65 | 64 | 87.84 | 1.35 | | 60 | Yes |
| FMT_control_Bacteria_bin.21_2 | Uninoc_bin21 | NA | 64 | 64 | 86.49 | 0 | | 61 | Yes |
| FMT_control_Bacteria_bin.59 | Uninoc_bin59 | NA | 67 | 67 | 90.54 | 0 | | 63 | Yes |
| FMT_control_Bacteroidetes_bin.11 | Uninoc_bin11 | NA | 69 | 69 | 93.24 | 0 | | 68 | Yes |
| FMT_control_Bacteroidetes_bin.23 | Uninoc_bin23 | NA | 68 | 68 | 91.89 | 0 | | 67 | Yes |
| FMT_control_Bacteroidetes_bin.26_2 | Uninoc_bin26 | NA | 66 | 66 | 89.19 | 0 | | 64 | Yes |
| FMT_control_Bacteroidetes_bin.5_2 | Uninoc_bin5 | NA | 66 | 65 | 89.19 | 1.35 | | 64 | Yes |
| FMT_control_Clostridiales_bin.22_2 | Uninoc_bin22 | NA | 65 | 62 | 87.84 | 4.05 | | 58 | Yes |
| FMT_positive_Bacteroidales_bin.11 | Inoc_bin11 | NA | 44 | 43 | 59.46 | 1.35 | | 39 | Yes |
| FMT_positive_Bacteroidales_bin.2_2 | Inoc_bin2 | NA | 66 | 66 | 89.19 | 0 | | 62 | Yes |
| FMT_positive_Bacteroidetes_bin.19_2 | Inoc_bin19 | NA | 60 | 59 | 81.08 | 1.35 | | 54 | Yes |
| FMT_positive_Bacteroidetes_bin.32 | Inoc_bin32 | NA | 44 | 43 | 59.46 | 1.35 | | 39 | Yes |
| FMT_positive_Deltaproteobacteria_bin.7 | Inoc_bin7 | NA | 70 | 57 | 94.59 | 17.57 | | 46 | Yes |
| Inoc_bin.75 | Inoc_bin75 | NA | 69 | 69 | 93.24 | 0 | | 65 | Yes |
| Uninoc_bin.2 | Uninoc_bin2 | NA | 67 | 67 | 90.54 | 0 | | 65 | Yes |
| Uninoc_bin.33 | Uninoc_bin33 | NA | 72 | 68 | 97.3 | 5.41 | | 57 | Yes |

**Table S1**. Statistics for all MAGs recovered in this study.

**#Limma-Voom with TMM normalization**OTU logFC AveExpr t P.Value adj.P.Val B

**Otu110** 2.596478 8.164939 4.036542 5.457194e-05 3.240734e-04 1.50605771

**#Ancom-BC**

Species beta se W p_val q_val diff_abn

**Otu110** 0.899298587 0.2999871 2.99779066 0.000000e+00 0.000000e+00 TRUE

**Taxonomy Note:**

OTU110:

Archaea(100);Euryarchaeota(100);Methanobacteria(100);Methanobacteriales(100);Methanobacteriaceae(100);Methanobrevibacter

**Table S2**. Summary of various differential enrichment tests based on both linear and negative binomial models showing the enrichment of **OTU110**, the only high abundance Euryarchaeal lineage in our dataset.
